# Delayed Migration and Perforation of the Jugular Vein by a Peripherally Inserted Central Catheter

**DOI:** 10.5811/cpcem.2017.9.35829

**Published:** 2017-10-18

**Authors:** Joshua J. Oliver, R. Erik Connor, Jacob R. Powell, Jessica M. Oliver, Brit Long

**Affiliations:** * San Antonio Uniformed Services Health Education, Department of Emergency Medicine, Fort Sam Houston, Texas; † Baptist Health System at Mission Trails, Intensive Care Unit, Department of Critical Care Nursing, San Antonio, Texas

## Abstract

We report a case of peripherally inserted central catheter (PICC) migration and perforation of the left internal jugular vein in a home health setting in an 80-year-old female. A left sided PICC was placed for treatment of diverticulitis following hospital discharge. She complained of sudden onset left sided neck pain immediately after starting an infusion of vancomycin. In the emergency department the injury was identified by portable chest radiograph and computed tomography of her neck. Following removal of the line, she had an uneventful course. Emergency physicians should be aware of this possible PICC line complication.

## INTRODUCTION

The use of peripherally inserted central catheters (PICC) has become nearly ubiquitous in patients requiring long-term intravenous (IV) access. Many studies have been published describing the possible complications of these lines. One prospective study described migration, infection, and obstruction as complications after 4,349 patient-days of observed PICC line use.^1^ Another discusses PICC line fracture and local venous thrombosis.^2^ Other studies mention perforation as a risk, but only in the large vessels of the upper arm most commonly used for PICC sites: the cephalic, basilic, and brachial veins. While neither study provides numbers for their assertions, they suggest the most common causes of perforation are the initial insertion itself and erosion of the vessel wall after long-term use.^3^,^4^

Several studies have attempted to determine which arm is ideal; however, neither the left or right arm has demonstrated a decreased risk of adverse events nor increased ease of access.^5^,^6^ Indeed, standards of practice published by an infusion nursing society suggest that factors such as pain, overlying infection, and previous surgeries (e.g. mastectomy with lymph node dissection) should be considered when selecting the ideal site.^7^ Regarding migration of PICC lines, two studies have documented as much as nine millimeters (mm) of movement of the tip with respiration and 21 mm of movement of the PICC line tip with abduction and adduction of the arm.^4^,^8^ Given these factors, it is not surprising that another author would conclude that even with ideal placement of a PICC line tip in the middle of the superior vena cava (mid SVC), said placement may not be protective against migration.^9^

The following case details a PICC line that migrated from the SVC and perforated the internal jugular vein (IJV) in the setting of home health. A literature search of Pubmed, Medline, and Ovid using the search “peripherally inserted central catheter perforation jugular vein” yielded no similar results.

## CASE REPORT

**Image 1:**
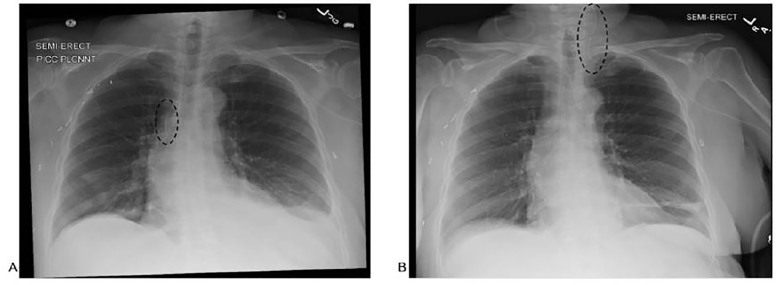
Portable chest radiographs demonstrating (A) initial placement of the left sided peripherally inserted central catheter in the middle superior vena cava; and (B) migration of the line to the left internal jugular vein (circles).

An 80-year-old women with a distant history of right breast cancer status post right mastectomy and lymph node dissection presented to the emergency department (ED) complaining of left-sided neck pain via ambulance from home health. Eleven days prior she had been discharged from the hospital after several days of treatment of IV vancomycin for newly diagnosed diverticulitis. The day she was discharged, a PICC line was placed in her left arm, with placement verified in the SVC via portable chest radiograph (Image 1a). The PICC continued to be used by home health for the intervening 11 days until she immediately complained of left-sided neck pain at the onset of vancomycin infusion. At the onset of pain, the patient described hearing a “whooshing” sound in her left ear. She did not complain of any numbness or weakness, and none was appreciated on her exam.

**Image 2:**
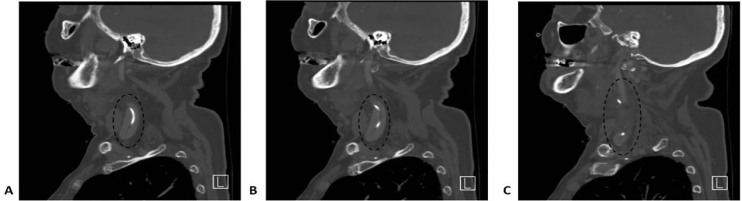
Sagittal computed tomography with contrast of the patient’s left neck (A superficial to C deepest) demonstrating perforation of the internal jugular vein with the peripherally inserted central catheter (circles)

In her initial evaluation, a portable chest radiograph was obtained that demonstrated line migration (Image 1b). This migration was confirmed by computed tomography (CT) of the neck, which also demonstrated that the PICC had perforated her IJV. The CT also revealed mass effect on the hypopharyngeal airway due to extravasation of vancomycin, resulting in severe narrowing and rightward shift of the airway (Images 2a–c). However, at no time did the patient appear to be in respiratory distress or any other kind of extremis.

Following admission, the PICC line was removed under supervision of an interventional radiologist, although no special equipment or maneuvers were required. The patient finished her course of IV vancomycin in the hospital and was subsequently discharged without event. On follow-up she had no lingering neck complaints and had not developed any sequelae.

## DISCUSSION

Home healthcare continues to be a popular option because of patient preference and decreased cost.^10^ In this setting the PICC line is often a preferred form of IV access because it does not require a physician or an operating room to place, and unlike a peripheral or midline IV, vesicant or irritant medications can be delivered through it.^7^

The popularity of PICC lines in the home health setting demonstrates the likelihood that emergency physicians will be faced with the reality of identifying complications associated with these devises. This case in particular highlights a previously undocumented complication of PICC lines. If a provider suspects his patient’s PICC line has migrated to the IJV and possibly perforated it, imaging that encompasses the upper arm and chest such as a portable chest radiograph, as well as computed tomography imaging of the neck is prudent. Further, interventional radiology and vascular surgery consults may be indicated depending on the type and severity of the complication.

**CPC-EM Capsule**

What do we already know about this clinical entity?

It is known that peripherally inserted central catheters (PICC) can migrate after insertion.

What makes this presentation of disease reportable?

This case documents a left sided PICC that migrated into the left internal carotid artery and subsequently perforated the artery.

What is the major learning point?

At the onset of an antibiotic infusion via the PICC the patient complained of left sided neck pain, which led the ED team to investigate.

How might this improve emergency medicine practice?

Recognition of this constellation of symptoms could aid future providers in early identification of this PICC line complication.

## CONCLUSION

Peripherally inserted central catheters are a popular option in the home healthcare setting for multiple reasons; however, their use is not without risk. Many of the complications associated with PICC lines have been well documented in the literature. This case describes a novel complication whereby a PICC line migrated from the SVC to the left IJV, resulting in subsequent perforation and extravasation of vancomycin in a home healthcare setting. It would be prudent for any provider who encounters a similar patient to obtain imaging and specialty consultation as appropriate.
